# Aspirin Exerts Its Antitumor Effect in Esophageal Squamous Cell Carcinoma by Downregulating the Expression of ATAD2 and KIF4A

**DOI:** 10.1155/2022/7005328

**Published:** 2022-08-21

**Authors:** Man Zhang, Zhenzhen Ren, Xianzeng Wang, Cong Liu, Zhaoyang Zheng, Junwei Zhao, Hongchun Liu

**Affiliations:** ^1^Department of Medical Laboratory, The First Affiliated Hospital of Zhengzhou University, Key Clinical Laboratory of Henan Province, Zhengzhou, Henan Province, China; ^2^Department of Medical Laboratory, People's Hospital of Zhengzhou, Zhengzhou, Henan Province, China; ^3^Department of Thoraric Surgery, Linzhou People's Hospital, Linzhou, Henan Province, China; ^4^Department of Medical Laboratory, The Second Affiliated Hospital of Henan University of Traditional Chinese Medicine, Zhengzhou, Henan Province, China; ^5^Department of Medical Laboratory, The First Affiliated Hospital of Zhengzhou University, Key Clinical laboratory of Henan Province, No. 1, Jianshe East Rd., Zhengzhou, 450000 Henan Province, China

## Abstract

**Objective:**

To investigate the expression of ATPase family AAA domain-containing protein 2 (ATAD2) and kinesin family member 4A (KIF4A) in esophageal squamous cell carcinoma (ESCC) tissues and their association with clinicopathological features and to explore the role of ATAD2 in regulating KIF4A expression and biological functions in ESCC cells and the effect of aspirin on their expression.

**Methods:**

The mRNA and protein expression of ATAD2 and KIF4A in the tissues of patients with ESCC were measured by RT-qPCR and immunohistochemistry, and the correlation between the expression of mRNA and clinicopathological characteristics was analyzed. Western blot and RT-qPCR were used to detect the interference efficiency and KIF4A expression after si-ATAD2 transfection in EC109 and KYSE30 cells. CCK-8 and Transwell assay were performed to investigate the effects of ATAD2 and aspirin on proliferation, migration, and invasion of ESCC cells. The effect of aspirin on the expression of ATAD2 and KIF4A in ESCC cells was measured by RT-qPCR and Western blot.

**Results:**

The expression of ATAD2 and KIF4A was upregulated in ESCC tissues, and both were correlated with the differentiation grades and lymph node metastasis. Knockdown of ATAD2 in ESCC cells significantly inhibited cell proliferation, migration, and invasion. Compared to the negative control group, the proliferation, migration, and invasion ability of ESCC cells in the aspirin-treated groups were decreased, and the expression of ATAD2 and KIF4A in ESCC cells was decreased after treating with aspirin for 48 h.

**Conclusion:**

The expression levels of ATAD2 and KIF4A are elevated in ESCC. ATAD2 promotes proliferation, migration, and invasion of ESCC cells by regulating KIF4A. Aspirin can inhibit the malignant behavior of ESCC cells by downregulating ATAD2 and KIF4A.

## 1. Introduction

Esophageal cancer is one of the gastrointestinal malignancies with high morbidity and mortality because of its insidious symptoms, high malignancy, and powerful evasiveness [1–3]. Esophageal squamous cell carcinoma (ESCC) is the most common histological type [4], which is characterized by advanced diagnosis, metastasis, drug resistance, and frequent recurrence, and more than 50% of patients have unresectable tumors or metastatic lesions at the time of diagnosis [5]. Although the progresses of diagnosis and treatment technology have greatly improved the prognosis of ESCC patients in recent decades, the 5-year survival rate remains low at approximately 15-20% [4, 6].

ATPase family AAA domain-containing protein 2 (ATAD2), also known as AAA+ nuclear coregulatory cancer-associated protein (ANCCA), is a member of the AAA+ ATPase family [7, 8]. It contains two domains, ATPase-binding site and bromine domain. Such structural feature of ATAD2 provides theoretical support for its functional activity as a coregulatory factor [9]. Studies have found that ATAD2 plays an important role in the progression of various tumors, including breast cancer [7, 10], lung cancer [11, 12], prostate cancer [10], hepatocellular carcinoma [13], and cervical cancer [14], and is involved in regulating tumor cell growth, migration, differentiation, cell cycle, and apoptosis [15]. Kinesin family member 4A (KIF4A), a member of the kinesin superfamily (KIFs), which was firstly identified by Ronald et al. on the axoplasm from squid giant axon and highly conserved in all eukaryotes [16]. KIF4A is involved in multiple cellular activities, particularly spindle formation and centrosome assembly in mitosis, chromosome concentration and separation, and DNA damage repair [17]. In addition, KIF4A is overexpressed in a variety of tumors [18]. A study on breast cancer established that ATAD2 could be recruited to the KIF4A promoter region by estrogen receptors alpha (ER*α*) and other transcription factors to increase the transcription of KIF4A [19]. These findings suggest that KIF4A may have functions that contribute to abnormal cell function and cancer progression.

Aspirin, a kind of widely used nonsteroidal anti-inflammatory drug (NSAIDs), is mainly used for pain relief, anti-inflammatory, and anticoagulation [20]. Emerging epidemiological evidence indicates that long-term low-dose aspirin use reduces the risk of tumor incidence and metastasis, including those of esophageal cancer and colorectal cancer, and therefore, it can be used in combination with antitumor therapy [21, 22]. Aspirin plays an important role in inhibiting tumor cell proliferation, metastasis, and drug resistance. Possible pharmacological mechanisms of aspirin include inhibition of the cyclooxygenase (COX) pathway, or COX-independent mechanisms, such as the PIK3CA pathway and Wnt/*β*-catenin pathways or treatment-induced cancer cell senescence [23, 24]. However, little is known about its specific function and potential mechanism in ESCC and its relationship with ATAD2 and KIF4A. Based on the above mentioned, we propose that aberrant expression of ATAD2 in ESCC could be a new therapeutic target and that aspirin exerts its antitumor activity in ESCC cells by inhibiting the expression of ATAD2 and KIF4A. Therefore, we carried out a series of experiments to demonstrate the role of ATAD2 and KIF4A and the antitumor effects of aspirin in ESCC.

## 2. Materials and Methods

### 2.1. Ethics Statement

All procedures performed in studies involving human participants were in accordance with the ethical standards of the institutional and/or national research committee and with the 1964 Helsinki Declaration and its later amendments or comparable ethical standards. This study was approved by the ethics committee of the First Affiliated Hospital of Zhengzhou University (Approval No.2021-KY-1131-002), and all participants signed informed consent documentation.

### 2.2. Patients and Tissue Samples

The pathological tissues used in this study were obtained from the First Affiliated Hospital of Zhengzhou University and included 50 pairs of cancer tissues and adjacent normal tissues of ESCC patients. The inclusion criteria for ESCC patients were as follows: (1) they were pathologically diagnosed with ESCC; (2) no prior radiation or chemotherapy; (3) the patient had no serious perioperative complications; (4) the patient had no other cancer except esophageal squamous carcinoma; (5) clinical case data was complete.

### 2.3. Cell Lines and Cell Culture

The human ESCC cell lines EC109 and KYSE30 were purchased from the Chinese Academy of Science Cell Bank (Shanghai, China). The cell lines were cultured in our research laboratory and grown in RPMI 1640 medium (HyClone, USA) supplemented with 10% fetal bovine serum (Gibco, USA) at 37°C with 5%CO_2_.

### 2.4. RNA Extraction and Reverse Transcription Quantitative Polymerase Chain Reaction

Total RNA was isolated using TRIzol reagent (TaKaRa, Dalian, China) and then reverse-transcribed the mRNA using the PrimeScript™ RT reagent Kit with gDNA Eraser (TaKaRa, Dalian, China) according to the manufacturer's instructions. The mRNA expression levels were determined by fluorescent quantitative PCR using SYBR® Premix Ex Taq™ II (TaKaRa, Dalian, China) and analyzed on the LightCycler 480 II Real-Time PCR System (Roche, Switzerland). GAPDH was used as an internal control for the mRNA expression analysis. The relative mRNA expression levels of ESCC tissues were determined using 2^-*Δ*Ct^ method, and the relative mRNA expression levels of cells were determined using 2^-*ΔΔ*Ct^. The primers for qPCR were designed and synthesized by Sangon Biotech (Shanghai) Co., Ltd (Table 1).

### 2.5. Immunohistochemistry

Each paraffin-embedded tissue specimen was at a thickness of 4 *μ*m. The operating procedures were described before. The paraffin-embedded slides were dewaxed in xylene and rehydrated in a graded concentration alcohol. Antigen retrieval was then performed by immersing slides in citrate-EDTA buffer and microwaving 2 min at high power and 20 min at low power. After blocking the endogenous peroxidases by incubation with 3% H_2_O_2_ for 20 min at room temperature, nonspecific immunoglobulin binding was blocked using 10% goat serum in PBS. The slides were incubated with ATAD2 antibody (dilution 1 : 200, Abcam, UK), KIF4A antibody (dilution 1 : 200, Proteintech, USA) at 4°C overnight. On the next day, the slides were incubated with biotin-conjugated secondary antibodies for 30 minutes followed by incubation with streptavidin–biotin conjugated with HRP. The slides were then stained with DAB for 2 min. Nuclei were stained with hematoxylin, dehydrated with gradient ethanol, cleared with xylene, and finally sealed with neutral gum. The staining was scored by two experienced pathologists. The final score was determined by combining the staining intensity score (0, negative; 1, weak; 2, strong) and the score of the proportion of positively stained tumor cells (0, 0%; 1, 1–50%; 2, 51–75%; 3, >75%). The final score of each sample ranged from 0 to 6.

### 2.6. Small Interfering RNA (siRNA) Transfection

The sequences of small interfering RNA (siRNA) targeting ATAD2 and si-NC were constructed from RiboBio (Guangzhou, China) (Table 2). 2 × 10^5^ cells were plated per well in six-well plates, and the degree of cell fusion reached 30%-40% on the next day. EC109 and KYSE30 cell lines were then transfected using riboFECT™ CP Reagent (RiboBio, Guangzhou, China) according to the manufacturer's instructions. We transfected siRNA targeting ATAD2 at a concentration of 50 *μ*M. Cells were then collected 48 h after transfection for subsequent experiments.

### 2.7. Western Blot Assay

Total cell proteins were extracted using RIPA lysis buffer and protease inhibitor (CoWin Biosciences, Inc., Beijing, China). The protein concentration was measured with a BCA Protein Assay Kit (CoWin Biosciences, Inc., Beijing, China), and the samples were diluted to the same concentration using RIPA lysis buffer. After boiling for 10 min, the samples were diluted with 4× loading buffer (Beyotime, China). Equal protein amounts of the samples were separated by 10% SDS-PAGE and transferred on PVDF membranes (Millipore, Billerica, MA, USA). Then the membranes were blocked with 5% nonfat skim milk, followed by incubating with primary antibody ATAD2 (dilution 1 : 1000, Abcam, UK), KIF4A (dilution 1 : 1000, Proteintech, USA), and GAPDH (dilution 1 : 1000, Proteintech, USA) at 4°C overnight. After incubating with HRP-conjugated antirabbit IgG secondary antibody (dilution 1 : 5000, Bioss, Beijing, China) for 1 h at room temperature, the protein bands were visualized with eECL Western Blot Kit (Beyotime, Beijing, China), and then the protein bands were developed.

### 2.8. Cell Proliferation Assay

The proliferation ability of EC109 and KYSE30 cells was measured by Cell Counting Kit-8 (Dojindo Laboratory, Japan). ESCC cells were digested using trypsin after transfection for 24 h, centrifuged, and resuspended in culture medium. Cells then were counted under microscope, diluted to 30000 cells/ml, and added to each well of a 96 well plate with 100 *μ*l cell suspension. Add CCK-8 reagent at 24 h, 48 h, 72 h, and 96 h, respectively, and leave to incubate for 2 h, and then the absorbance value was detected at wavelength 450 nm using enzyme standard.

### 2.9. Migration and Invasion Assays

The migration and invasion assays were conducted using Transwell insert chambers (8 *μ*m pore size, Corning, NY, USA) with or without matrigel. For the migration assay, 3 × 10^4^ transfected cells suspended in 200 *μ*l of serum-free 1640 media were added to the upper chamber, and 600 *μ*l RPMI 1640 supplemented with 15% FBS was added into the lower chamber, while for the invasion assay, the membrane of upper chamber was coated with 300 ng/ml matrigel (BD Biosciences, USA) except for the above conditions. After incubation for 24 h at 37°C, cells remaining on the top surface of the chamber were removed with a cotton swab, followed by fixing the cells on the lower surface of the membrane through 4% paraformaldehyde and staining by crystal violet. The cells were subsequently counted in three independent fields by microscope.

### 2.10. Aspirin Treatment

Four groups of aspirin (Solarbio, Beijing, China) solution with different concentrations were prepared: negative control group (0 mmol/l), low concentration group (0.5 mmol/l), medium concentration group (2.5 mmol/l), and high concentration group (5 mmol/l). EC109 cells and KYSE30 cells in logarithmic growth stage were obtained. The cells were resuspended with medium containing different concentrations of aspirin and seeded into the culture plate. Other steps are the same as described above.

### 2.11. Statistical Analysis

The SPSS 21.0 software (SPSS Inc., Chicago, IL, USA) and GraphPad Prism 7 (GraphPad Software Inc., La Jolla, CA, USA) were used to analyze the experimental data. Statistical analysis was performed using independent samples *t*-test for data with two independent samples that conformed to normal distribution, rank sum test for data with two independent samples that did not conform to normal distribution, one-way analysis of variance for comparison of quantitative data among multiple groups, and *chi*-square test for statistical analysis of clinicopathological parameters in categorical data, with *P* < 0.05 considered statistically significant.

## 3. Results

### 3.1. Differential Expression of ATAD2 and Their Relationship with Clinicopathological Features

**Figure 1:**
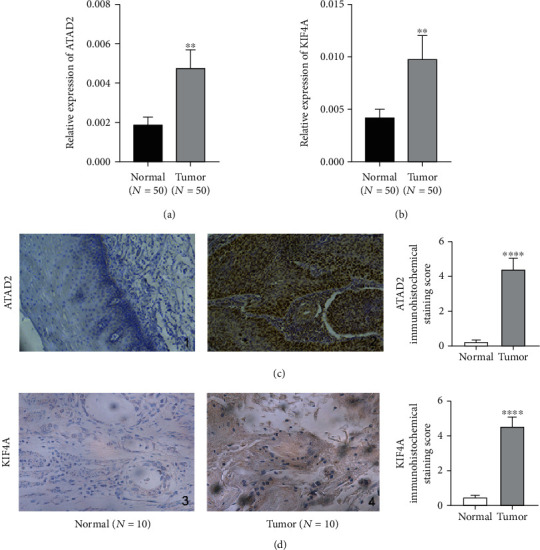
ATAD2 and KIF4A are highly expressed in ESCC. (a) ATAD2 in normal esophageal squamous epithelium and ESCC tissues was measured by RT-qPCR. (b) Highly expressed KIF4A in esophageal squamous epithelium and ESCC was measured by RT-qPCR. (c) Immunohistochemical results showed that ATAD2 protein was highly expressed in cancer tissues. (d) A highly level of KIF4A protein expression in ESCC tissues was revealed by immunohistochemical results. ^∗∗^*P* < 0.01.

The mRNA and protein expression of ATAD2 and KIF4A in ESCC tissues was significantly higher than that in adjacent normal tissues (*P* < 0.01) (Figures 1(a) and 1(b)). Immunohistochemistry staining showed that ATAD2 was mainly located in the nucleus of ESCC cell and KIF4A is localized both in the cytoplasm and nucleus, and their protein expression level was higher in ESCC (Figure 1(c)). Based on the median values of the relative mRNA expression levels of ATAD2 and KIF4A in 50 ESCC tissues, the samples were divided into low and high expression groups, respectively. The associations of ATAD2 and KIF4A with ESCC patient clinical outcome were analyzed, including age, gender, tumor size, tumor differentiation, and distant lymph node metastasis. Results are shown in Table 3. We found that the ATAD2 level was correlated with the differentiation grade (*P* < 0.0001) and lymph node metastasis (*P* = 0.0016); however, there was no significant difference between ATAD2 level and sex, age, or tumor size. The expression level of KIF4A was significantly correlated with the grade of ESCC differentiation (*P* = 0.0227) and lymph node metastasis (*P* = 0.0016), but not with patient sex, age, and tumor size (*P* > 0.05).

### 3.2. Determination of ATAD2 Interference Efficiency and KIF4A Expression after siRNA Transfection into ESCC Cells

**Figure 2:**
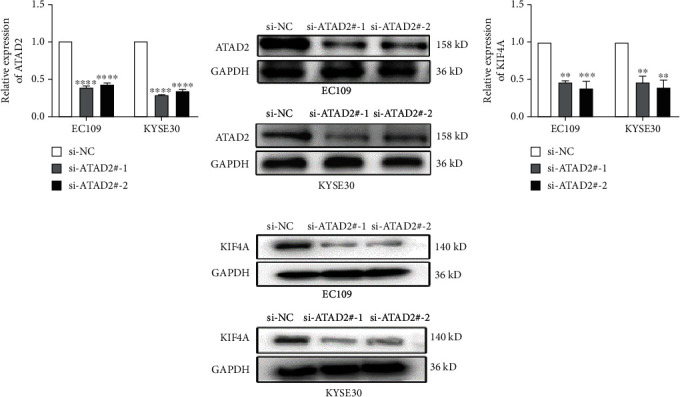
ATAD2-regulated KIF4A expression in ESCC cells. (a and b) The ATAD2 expression of si-ATAD2#1 and si-ATAD2#2 groups were significantly decreased compared with that of si-NC group in both mRNA and protein levels. (c and d) The KIF4A expression levels in both mRNA and protein level of EC109 and KYSE30 cells were suppressed in si-ATAD2#1 and si-ATAD2#2 groups. ^∗∗^*P* < 0.01, ^∗∗∗^*P* < 0.001, and ^∗∗∗∗^*P* < 0.0001.

To detect the effect of ATAD2 on ESCC cells, two siRNAs were constructed and transfected to EC109 and KYSE30 cells. The ATAD2 siRNA interference efficiency was observed to be markedly decreased both in mRNA and protein levels compared to negative control group by RT-qPCR and western blot (Figures 2(a) and 2(b)), respectively. After interfering with ATAD2 expression, the expression of KIF4A was further detected, and the results showed that the expression levels of KIF4A mRNA and protein in si-ATAD2 group were lower than that in negative control group (Figures 2(c) and 2(d)).

### 3.3. ATAD2 Promoted Proliferation Ability in ESCC Cells

**Figure 3:**
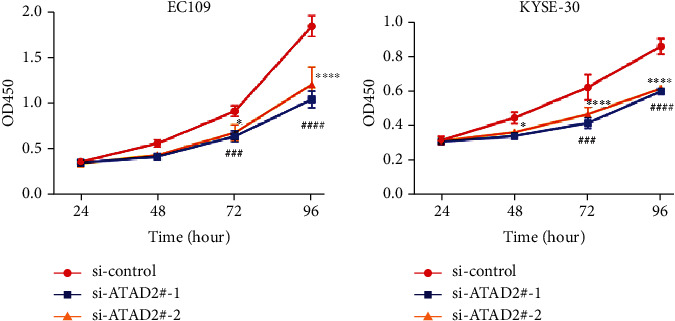
ATAD2 silencing reduces the proliferation ability of ESCC cells. (a and b) Cell proliferation rate evaluated by CCK-8 proliferation analysis. The two-way *ANOVA* test was adopted for comparison among multiple groups, followed by *Dunn's* multiple-comparison test. ^∗^*P* < 0.05, ^∗∗∗^, ###*P* < 0.001, ^∗∗∗∗^, ####*P* < 0.0001.

CCK-8 analysis was performed to detect the proliferation ability of EC109 and KYSE30 cells after transfection. The result showed that the knockdown of ATAD2 suppressed the proliferation ability of EC109 and KYSE30 cells compared with the siRNA negative control (Figures 3(a) and 3(b)).

### 3.4. ATAD2 Knockdown Inhibited the Migration and Invasion of ESCC Cells

**Figure 4:**
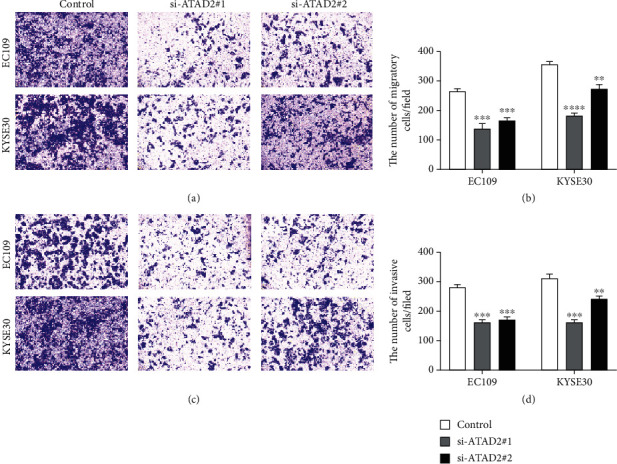
Migration and invasion assay in ESCC cell lines. (a, c) Transwell assay was performed to detect the effect of ATAD2 on the (a) migration and (c) invasion abilities in EC109 and KYSE30 cell lines. (b, d) ATAD2 knockdown inhibited the migratory and invasive abilities of EC109 and KYSE30 cells. ^∗∗^*P* < 0.01 and ^∗∗∗^*P* < 0.001.

To study whether ATAD2 could influence the migration and invasion capacities of ESCC cells, we performed cell migration and invasion assay after transfection with siRNAs targeting ATAD2. We found that ATAD2 knockdown resulted in significant inhibition of ESCC cell migration (Figures 4(a) and 4(b)). Furthermore, similar results were yielded in the cell invasion assay; the invasion ability of EC109 and KYSE30 cells was suppressed after transfection (Figures 4(c) and 4(d)).

### 3.5. The Proliferation of ESCC Cells Decreased after Aspirin Treatment

**Figure 5:**
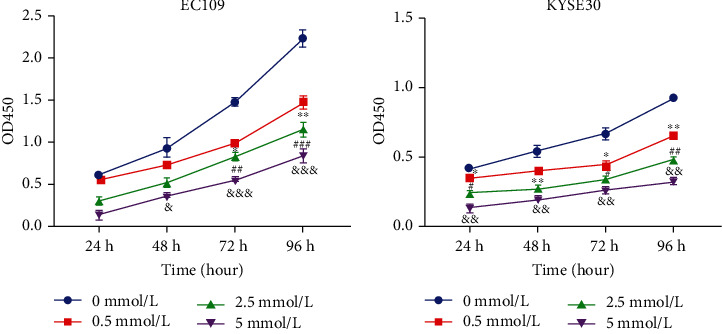
Effect of aspirin stimulation on proliferation of  ESCC cells. (a and b) CCK-8 proliferation analysis was performed to assess the impact of aspirin on the proliferation of ESCC cells. ^∗^, #, &*P* < 0.05, ^∗∗^, ##, &&*P* < 0.001, ###, &&&*P* < 0.0001.

After EC109 and KYSE30 cells were stimulated with different concentrations of aspirin for 24 h, 48 h, 72 h, and 96 h, cell proliferation activity was detected by CCK-8. Results as shown in the figure, aspirin significantly inhibited the proliferation rate of EC109 and KYSE30 in the concentration range of (0.5-5) mmol/l, and the inhibition became more obvious with the increase of drug concentration (Figures 5(a) and 5(b)).

### 3.6. The Migration and Invasion of ESCC Cells Decreased after Aspirin Treatment

**Figure 6:**
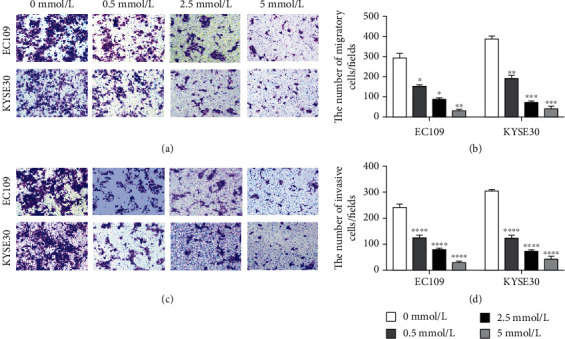
Effect of aspirin stimulation on migration and invasion of  ESCC cells. (a, c) Transwell assay was performed to detect the effect of aspirin on the (a) migration and (c) invasion abilities in EC109 and KYSE30 cell lines. (b, d) Treatment with aspirin inhibited the migratory and invasive abilities of EC109 and KYSE30 cells. ^∗∗∗∗^*P* < 0.0001.

After aspirin treatment, the migration and invasion ability of EC109 and KYSE30 were determined by Transwell assay. The results showed that the migration and invasion ability of EC109 and KYSE30 reduced after aspirin treatment and along with increasing concentration of aspirin in sequence (Figure 6).

### 3.7. The Expression of ATAD2 and KIF4A mRNA Decreased in ESCC Cells after Aspirin Stimulated

**Figure 7:**
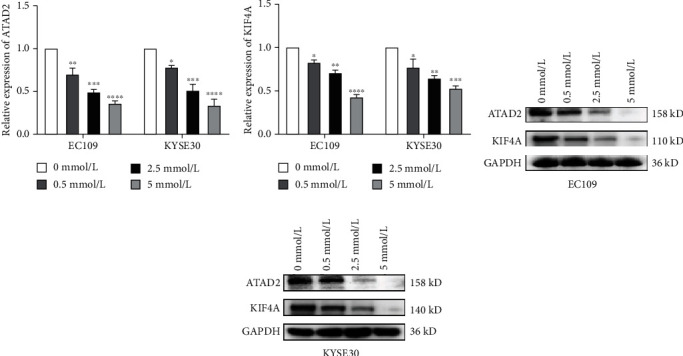
Effect of aspirin stimulation on mRNA expression levels ATAD2 and KIF4A. (a and b) Compared with aspirin 0 mmol/L group, the relative expression levels of ATAD2 and KIF4A mRNA in EC109 and KYSE30 cell lines decreased; (c and d) the protein expression levels of ATAD2 and KIF4A in EC109 and KYSE30 cell lines decreased. ^∗^*P* < 0.05, ^∗∗^*P* < 0.01, ^∗∗∗^*P* < 0.001, and ^∗∗∗∗^*P* < 0.0001.

After 48 h of stimulation with different concentrations of aspirin, compared with the negative control group, the relative mRNA and protein expression levels of ATAD2 and KIF4A mRNA in EC109 and KYSE30 cells decreased and sequentially decreased in turn with the increase of concentration. The results were shown in Figure 7, which showed that aspirin at different concentrations could reduce the expression of ATAD2 and KIF4A in ESCC cells (Figures 7(a)–7(d)).

## 4. Discussion

In recent years, with the development of medical diagnosis and treatment technology, the early diagnosis of ESCC has greatly improved [25, 26]. However, since many patients have no obvious early symptoms, the five-year survival rate remains unsatisfactory, and more effective treatment strategies are urgently proposed [27, 28]. Now, molecular targeted therapy is a rapidly developing field. Exploring the possible pathogenesis of ESCC and seeking new molecular targets are particularly important for improving the diagnosis and treatment of ESCC.

Because of its central role in the regulation of cellular activities, ATAD2 has been frequently reported in recent years. The AAA + ATPase domain of ATAD2 is involved in intracellular regulatory processes such as signal transduction, cell proliferation, and gene expression, whereas the bromodomain plays an important role in chromosome remodeling and transcriptional control of protein interactions [9, 15]. The structure of ATAD2 suggests that its function is closely related to genomic regulation and may be relevant to tumorigenesis. In addition, KIF4A provides power to the movement of intracellular organs such as microtubules and plays a key role in anaphase; its abnormalities can lead to abnormal mitotic checkpoints and DNA damage repair processes, as well as chromosomal instability and the formation of aneuploidy, which can cause cellular abnormal proliferation and differentiation leading to tumor formation [29, 30]. Studies have shown that ATAD2 could act as a coactivator to increase the transcription of KIF4A. Therefore, we speculated that ATAD2 might participate in the occurrence and development of ESCC by regulating KIF4A. Preliminary RT-qPCR and immunohistochemistry results of this study showed that ATAD2 and KIF4A in the paired ESCC tissues were highly expressed at both mRNA and protein levels compared with the adjacent normal tissues. In this study, ESCC cell lines EC109 and KYSE30 were cultured *in vitro*, and ATAD2 expression was knocked down by siRNA. The results showed that ATAD2 played an important role in the proliferation, migration, and invasion functions of ESCC cells. Also, the mRNA and protein levels of KIF4A were detected after depletion of ATAD2 in ESCC cells, and both were decreased compared with the negative control group. This suggested that highly expressed ATAD2 might promote tumorigenesis of ESCC by regulating the KIF4A expression.

At present, the study of the antitumor mechanism of aspirin is not in-depth. The proven mechanism is mainly its inhibition of cyclooxygenase-2 (COX-2) activity by irreversible acetylation; however, many studies have shown that aspirin is critical for cancer progression through other mechanisms independent of COX-2 [31, 32]. Whether it exerts antitumor activity and the mechanism on ESCC has little been reported. We found that after stimulation of EC109 and KYSE30 cells with different concentrations of aspirin *in vitro*, compared with the negative control group, the proliferation, migration, and invasion ability of ESCC cells treated with aspirin in the range of (0.5-5) mmol/l decreased with the increase of aspirin concentration. Meanwhile, the mRNA and protein levels of ATAD2 and KIF4A in EC109 and KYSE30 cells stimulated with different concentrations of aspirin for 48 h were both decreased, which suggested that aspirin might exert its antitumor activity in ESCC by inhibiting the expression of ATAD2 and KIF4A.

## 5. Conclusion

We have elucidated that the expression of ATAD2 and KIF4A was elevated in ESCC and effects of ATAD2 on proliferation, migration, and invasion of ESCC cells. Then we demonstrated that ATAD2 might regulate the carcinogenesis and development of ESCC by regulating KIF4A. In addition, we found that aspirin could exert antitumor effects in ESCC cells by inhibiting the expression of ATAD2 and KIF4A.These results suggested that ATAD2 correlated with the malignant status and could potentially serve as a therapeutic target in ESCC. Our study provided new theoretical support for antitumor combination therapy in clinical ESCC patients. ATAD2 could be proposed as a novel pharmacotherapeutic target for ESCC patients. However, the exact antitumor mechanism of aspirin still needs further study.

## Figures and Tables

**Table 1:** Primer sequences for RT-qPCR^a^.

Gene	Sequence (5′-3′)
ATAD2^b^	F^e^: CAACATATTTTATAGTGGCCCAGC
	R^f^: TCGTTTACAGTAAGGACTTCTTGGT
KIF4A^c^	F: TCTGTTTCAGGCTGCTTTCA
	R: GCCCTGAAATATTTGATTGGAG
GAPDH^d^	F: GAACGGGAAGCTCACTGG
	R: CCTGCTTCACCACCTTCT

^a^RT-qPCR: reverse transcription quantitative polymerase chain reaction; ^b^ATAD2: ATPase family AAA domain-containing protein 2; ^c^KIF4A: kinesin family member 4A; ^d^GAPDH: glyceraldehyde-3-phosphate dehydrogenase; ^e^F: forward; ^f^R: reverse.

**Table 2:** Sequences of siRNAs targeting ATAD2 and siRNA-NC.

siRNA^a^	Sequence (DNA)
si-ATAD2#-1	5′-GAAGTGCGTCGAAGTTGTA-3′
si-ATAD2#-2	5′-CGAACAGGCTAGATTCTAT-3′

^a^ siRNA: small interfering RNA.

**Table 3:** The relationship between the expression of ATAD2 and KIF4A and clinicopathological parameters in ESCC^a^ patients.

Clinical parameters	ATAD2		*P* value	KIF4A		*P* value
	High (25)	Low (25)		High (25)	Low (25)	
Sex						
Male	16	20	0.3451	18	18	0.9
Female	9	5		7	7	
Age						
<60	6	8	0.7536	5	9	0.3451
≥60	19	17		20	16	
Tumor size						
<3 cm	9	12	0.5672	8	14	0.1536
≥3 cm	16	13		17	11	
Differentiation grade						
G1	4	21	0.0001∗∗∗∗	8	17	0.0227∗
G2/G3	21	4		17	8	
Lymphatic metastasis						
N0	7	19	0.0016∗∗	7	19	0.0016∗∗
N1-N2	18	6		18	6	

^a^ESCC: esophageal squamous cell carcinoma; ^∗^*P* < 0.05; ^∗∗^*P* < 0.01; ^∗∗∗∗^*P* < 0.0001.

## Data Availability

The data used to support the findings of this study are available from the corresponding author upon reasonable request.
